# Minilaparoscopic Colorectal Resections: Technical Note

**DOI:** 10.1155/2012/482079

**Published:** 2012-03-27

**Authors:** S. Bona, M. Molteni, M. Montorsi

**Affiliations:** Department of General Surgery, IRCCS Istituto Clinico Humanitas, University of Milan School of Medicine, Via Manzoni 56, 20089 Rozzano, Milano, Italy

## Abstract

Laparoscopic colorectal resections have been shown to provide short-term advantages in terms of postoperative pain, general morbidity, recovery, and quality of life. To date, long-term results have been proved to be comparable to open surgery irrefutably only for colon cancer. Recently, new trends keep arising in the direction of minimal invasiveness to reduce surgical trauma after colorectal surgery in order to improve morbidity and cosmetic results. The few reports available in the literature on single-port technique show promising results. Natural orifices endoscopic techniques still have very limited application. We focused our efforts in standardising a minilaparoscopic technique (using 3 to 5 mm instruments) for colorectal resections since it can provide excellent cosmetic results without changing the laparoscopic approach significantly. Thus, there is no need for a new learning curve as minilaparoscopy maintains the principle of instrument triangulation. This determines an undoubted advantage in terms of feasibility and reproducibility of the procedure without increasing operative time. Some preliminary experiences confirm that minilaparoscopic colorectal surgery provides acceptable results, comparable to those reported for laparoscopic surgery with regard to operative time, morbidity, and hospital stay. Randomized controlled studies should be conducted to confirm these early encouraging results.

## 1. Introduction

Laparoscopic surgery (LS) for both benign and neoplastic colonic disease has become a standard procedure worldwide [1–8], although its distribution is currently limited [9]. Many authors reported adequacy and short-term benefits also for laparoscopic rectal procedures [10–13]; nevertheless, large randomized studies and oncologic results are still lacking. In recent years, innovative endoscopic procedures such as single-port laparoscopic surgery (SILS) [14], natural orifices transluminal endoscopic surgery (NOTES) [15], and needlescopic surgery (NS) [16] have been introduced to further reduce surgical invasiveness and abdominal wall trauma. This goal has been achieved by reducing the number of ports (SILS), avoiding transabdominal incisions (NOTES), or reducing port size (NS). This should possibly reduce postoperative pain and lower the incidence of wound infections and port site hernias, besides improving cosmetic results. NOTES has been performed mainly on experimental models [17, 18], and its application in clinical environment is very limited [19, 20]. Several attempts with single-port technique have been made for various procedures, including appendectomy [21], cholecystectomy [22], splenectomy [23], inguinal hernia repair [24], and in paediatric [25], gynaecologic [26], and urologic [27] surgery; few preliminary experiences are available also for colorectal surgery [28–46]. Likewise, NS has been gradually introduced in the aforementioned surgical fields, with some preliminary results also in colorectal surgery [47–55]. The main drawback of SILS is the loss of triangulation of surgical instruments in the operative field, which despite recent development of curved instruments and flexible endoscopes enhances technical difficulty and requires a long learning curve. Needlescopic technique keeps port positioning unchanged compared to standard laparoscopic procedures and therefore has minimal impact on the surgeon. Nevertheless, few technical aspects need to be considered when approaching needlescopic colorectal surgery. Since reports are limited in this field, we aim to review technical points such as instrumentation and its use in the different steps of the operation.

## 2. Instrumentation

**Figure 1:**
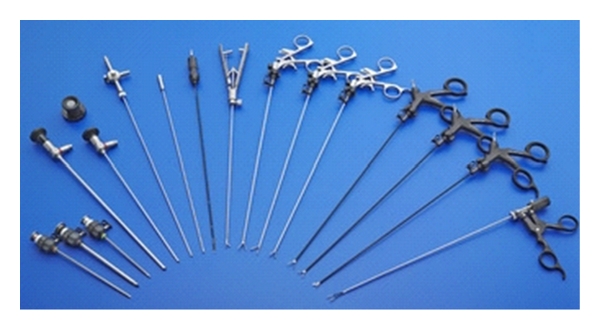
Minilaparoscopic 3 mm instrumentation available to date.

In our practice, laparoscopic colorectal resections are currently performed with a 3- to 5-port (5–12 mm size) technique, intracorporeal anastomosis whenever possible, and specimen extraction through a suprapubic transverse incision. Laparoscopic instrumentation consists of 30° scope, atraumatic graspers, coagulating hook, bipolar grasper, clip applier, ultrasonic dissector (optional), suction device, retractor, needle holder, and linear stapler. Apart from the clip applier, the ultrasonic dissector, and the stapler, all instruments are available in 3 mm size (Figure 1) still keeping a high standard of quality and performance. Only 3 mm laparoscopes, although providing a good vision, are still less performant than 5 mm HD scopes which may be preferable in advanced laparoscopic procedures. Since a minilaparotomy is always planned, open access with a Hasson port may be performed at the suprapubic site allowing introduction of 10–12 mm devices. Further trocars ranging from 3 to 5 mm size are placed after insufflation under direct vision.

## 3. Left Colectomy and Rectal Resection

**Figure 2:**
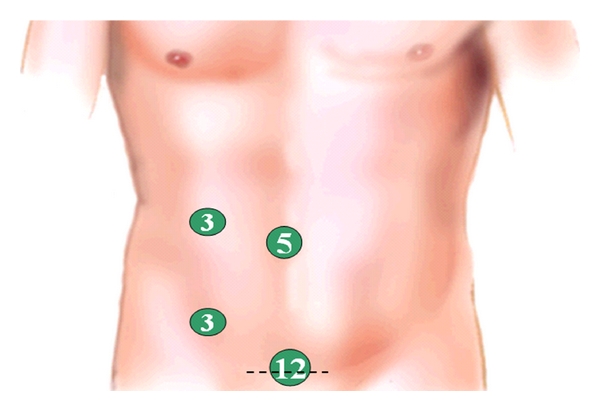
Trocar placement for left-side resection.

**Figure 3:**
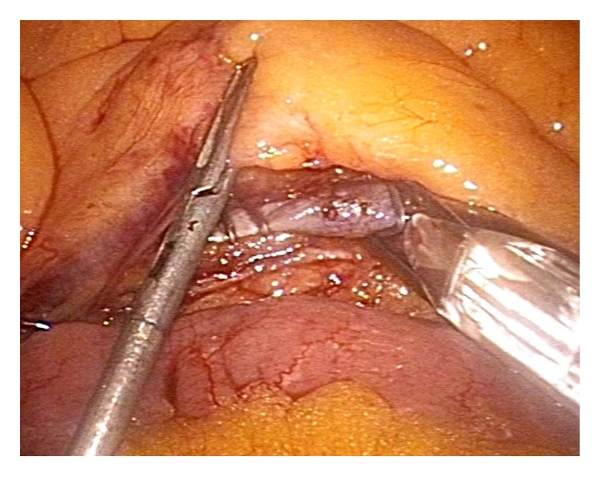
Three-millimeter grasper exposes IMV (3 mm port in the right hypochondrium, left hand) while 12 mm device places clips for vessel division (12 mm port above the pubis, right hand).

**Figure 4:**
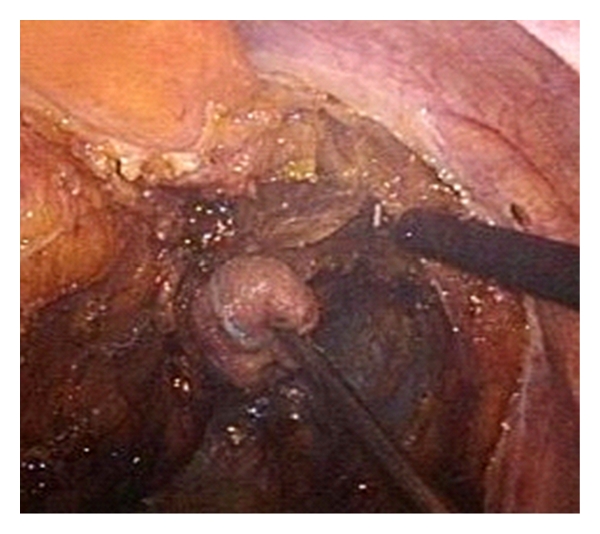
Dissection of the mesorectal right side.

**Figure 5:**
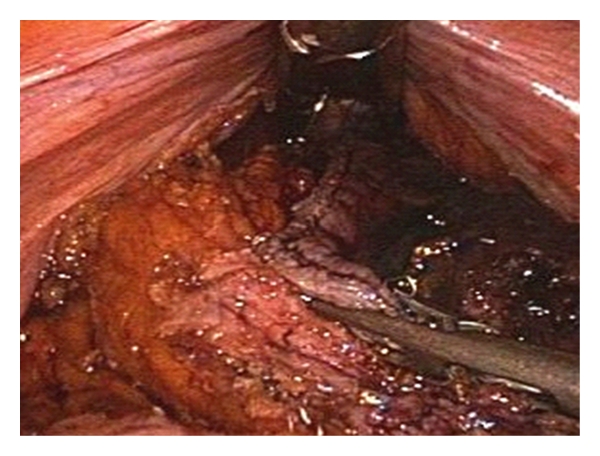
Rectal transection performed by linear stapler introduced by the suprapubic 12 mm port.

Port positioning for minilaparoscopic left colectomy is shown in Figure 2. After placement of the 12 mm Hasson port at the site of the planned minilaparotomy, one 5 mm port is inserted through the umbilicus for the scope, and two 3 mm ports are placed in the right hypochondrium on the midclavicular line and in the right lower quadrant. Such position allows good triangulation in order to work between the left hypochondrium and the pelvis. An additional 3 mm port may be placed in the left lower quadrant for the surgeon to switch hands and improve triangulation during mobilization of the splenic flexure or dissection of the lower rectum. When in place, this port may be used by the assistant for additional grasping or to expose the operative field with a retractor when working in the pelvis. A standard medial to lateral approach is used starting with vascular ligation followed by Toldt's fascia dissection. Clips for vascular ligation are inserted through the 12 mm suprapubic port (Figure 3). Mobilization of the splenic flexure may be performed indifferently as a first step or before bowel section. Dissection is performed with the 3 mm coagulating hook; should the ultrasonic dissector be used, the 3 mm port in the right and/or left lower quadrant is to be replaced with a 5 mm port. Three mm instruments allow fine grasping of elements such as vessels and peritoneum, but care must be taken during lifting of the mesocolon as the small contact surface may result in the tearing of the vessels which need to be preserved; it is therefore advisable to interpone a sponge (inserted through the 12 mm port) between the grasper and the tissue to be handled. Similarly, since mesorectal integrity is of utmost importance during total mesorectal excision in rectal cancer surgery, grasping of the mesorectal fascia with small instruments is to be avoided, and a wad of gauze held by the grasper should be used to expose the “holy plane” (Figure 4). If a stronger retraction is needed to achieve dissection of the lower rectum or in case of bulky tumours in obese patients, a 10 mm retractor may be introduced through the 12 mm suprapubic port. The same port is used to place the linear stapler and transect the rectum at any level down to the pelvic floor (Figure 5). After specimen retrieval, the suprapubic minilaparotomy is closed leaving in place the 12 mm port which may be useful for extraction of the staple trocar, anterior retraction during confection of low colorectal anastomosis, and introduction of sutures if the peritoneum is to be closed. Alternatively, the suprapubic minilaparotomy may be performed as a first step of the operation and sealed temporarily with a device which allows air-tight placement of a 12 mm port. At the end of the procedure, the ports are removed under vision to check eventual bleeding, and the 12 mm port is extracted at last.

## 4. Right Colectomy

**Figure 6:**
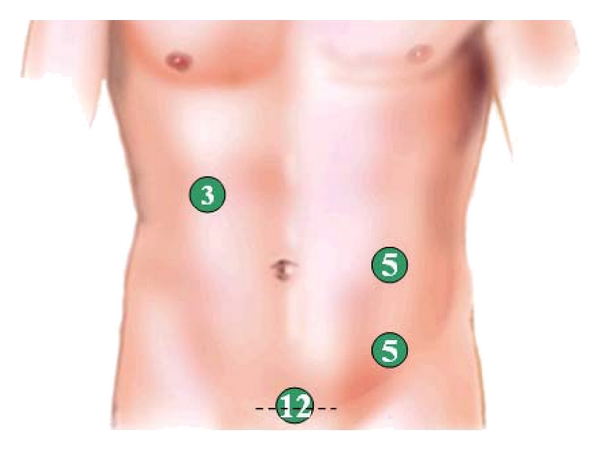
Trocar placement for right–side resection.

**Figure 7:**
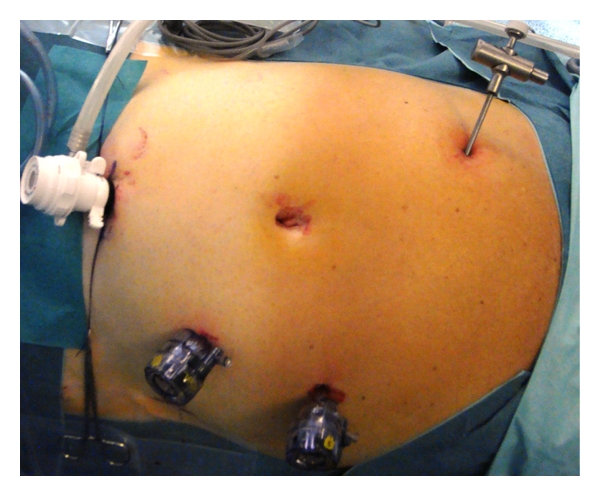
Trocar placement for right–side resection.

The 12 mm Hasson port is inserted above the pubis using the open technique, and two additional ports are placed under vision: one 5 mm port is placed in the left lower quadrant for the introduction of the scope and one 3 mm port in the left hypochondrium on the midclavicular line. Such position allows good triangulation when working in the right abdomen and on the middle transverse colon. The use of the ultrasonic dissector requires a 5 mm port in the left upper quadrant. An optional 3 mm port may be placed in the right hypochondrium to allow grasping and retraction by the assistant (Figures 6 and 7). Dissection is carried on with the same principles described above. The clip applier and linear stapler are introduced through the 12 mm port. After completing the mobilization and the bowel transaction, the specimen is pushed in the right hypochondrium. A double enterotomy is performed in the distal ileum and transverse colon, and a stapled side-to-side isoperistaltic anastomosis is performed. Due to the direction of the linear stapler introduced through the suprapubic port, the visceral stumps must be correctly oriented using one or two traction sutures held by graspers. The anastomosis is completed with a running suture, and the ileal mesentery and transverse mesocolon are approximated. Five and 3 mm ports are retrieved under vision, and the specimen is extracted via a suprapubic incision.

## 5. Discussion

Laparoscopy has been widely proven to be a feasible, safe, and effective technique to perform colorectal resections [1, 2, 56–61] leading to clinically relevant advantages in selected patients such as reduction of postoperative pain [1, 62] and complications, shortening hospital stay and improving recovery [1, 58, 63], wound healing [1, 64], and cosmesis [65, 66]. Moreover, minimally invasive surgery has facilitated the application of enhanced recovery programs in colorectal surgery [67–69]. Long-term outcome of laparoscopic colonic resection for cancer is not different from what has been achieved by open surgery procedures [2]. Therefore, some authors suggest that laparoscopy should be the preferred technique to perform colectomy in patients suitable for this approach [1]. New trends have been developed in order to further reduce the impact of surgical procedure in patients undergoing colorectal resections. Three main directions have been undertaken in specialized centres: SILS, which aims to the reduction of port number, NOTES, in which surgical instruments are inserted in hollow organs trough natural openings, and minilaparoscopic colorectal surgery, based on reduction of port size.

SILS was first described by Piskun and Rajpal for cholecystectomy as early as 1999 [14]; this term currently identifies surgical procedures that provide the placement of one port having three or more working channels within the umbilicus. Surgeons who perform single-port colorectal surgery seem to agree that this technique, though should be suitable for the resection of colon cancer with respect to oncologic principles, is demanding because of the difficulties of exposure of the operative field and because of the risk of “crowding” while maneuvering laparoscopic instruments, although specially designed for this purpose [44].

NOTES was first described by Kalloo et al. in 2004 [15]: this term currently identifies surgical procedures that provide the placement of flexible endoscopic systems through natural orifices (per-oral, transvaginal, transanal, transumbilical, or transvesical routes) entering the peritoneal cavity through an incision of hollow organs and approaching target organs to perform intra-abdominal procedures. Many procedures ranging in complexity from cholecystectomy to colorectal resections may be theoretically performed entirely endoscopically without the need for abdominal incisions [70, 71]. The advantages of such an approach include absence of incisional pain and wound complications (including infection and hernias), improved cosmetic results, and faster recovery. Although studies have shown the feasibility of an NOTES approach, significant constraints have been identified with the use of a flexible endoscopy platform, including a relative inability to apply off-axis forces, mechanical stability, inadequate triangulation, and limits in passing multiple instruments simultaneously into the peritoneal cavity. Concerns have also been expressed about the risk of postoperative leak and infections: with the intestinal closure systems currently adopted for NOTES access sites, it is doubtful that 100% safety can be achieved [72].

At present, the need for improved technology remains a major limitation for SILS and NOTES.

The use of smaller ports to perform laparoscopic procedures is defined with different terms such as “minilaparoscopy,” “microlaparoscopy,” “miniendoscopic” or “microendoscopic surgery,” and “microinvasive surgery” [16]. In general, NS is the term used to describe LS with instruments with an external diameter of 2-3 mm, as defined by Gagner and Garcia-Ruiz [16]. Santoro et al. have defined “miniendoscopic surgery” as any procedure that uses endoscopic instruments and optics 5 mm in diameter or smaller [55].

Needlescopic colorectal surgery is feasible, effective, and easy to perform since no specific training is required [55]. Surgeons who experienced NS in the aforementioned surgical fields [47–55] report several advantages over standard LS. In general, reduction of laparoscopic port size is associated with limited trauma on the abdominal wall. Smaller incisions result in decreased incisional pain and reduced risk of complications such as port-site bleeding, infection, and herniation. Moreover, minimal scarring allows better cosmetic results [73]. On the other hand, narrow operative field, lower image quality due to lack of definition and reduced light transmission [16, 74], and blurred vision with the use of electrocautery [75] are almost unanimously reported to be the “Achilles' heel” of this technique and cause more stress for the surgeon especially when using 3 mm scopes. The use of modern 5 mm optics with high-definition cameras and powerful light sources is much more comfortable in performing advanced laparoscopic procedures, though a 3 mm optic inserted through an ancillary port may be useful if the 5 mm port is to be used for a larger instrument such as the clip applier.

As for smaller instruments, they may show a weaker grasping capability and a lack of tensile strength due to increased flexibility, particularly in the presence of fibrosis or inflammation. Manipulation of tiny laparoscopic instruments may result in an increased risk of tissue damage during dissection [16, 74, 76–79].

Apart from these precautions, moving from standard laparoscopic technique to needlescopic colorectal resections is not to be considered as approaching a new technique but simply an adaptation of a well-established practice and does not require a long learning curve. None of the steps of the operation has shown difficulties resulting from the use of miniaturized instruments. A good exposition of the surgical field has been always achieved during vessel ligation and viscera dissection, transection, and anastomosis. Building on the experience gained from needlescopic procedures such as cholecystectomy and appendectomy, we decided not to give up the greater definition provided by 5 mm scopes, since the 3 mm optics are still less performant for more advanced and complex procedures.

The 3 mm grasper has been shown to provide good traction, also during gentle dissection. We used a simple trick to overcome its aforementioned limits: a wad of gauze held within the jaws of the instrument itself was used for lifting and retracting viscera in order to increase its strength and decrease the risk of injury of other organs.

One aspect that has been reconsidered performing needlescopic colorectal surgery is the position of trocars: we thought it would be logical to incorporate the only 12 mm port that must necessarily be placed for the introduction of the stapler in the minilaparotomy which is generally a transverse suprapubic incision; we therefore started introducing the stapler from a suprapubic port not only for low rectal resection but also to transect the upper rectum and transverse colon. The use of the stapler from the suprapubic port did not result in substantial differences in bowel transection. Nevertheless, performing an intracorporeal side-to-side mechanical ileocolic anastomosis from the suprapubic port requires wider mobilization of the transverse colon in order to place it parallel to the stapler. Approximation and orientation of the ileal and colonic stumps is best achieved by pulling on two stitches placed at each end of the anastomosis, the proximal one being held by the 3 mm grasper in the right hypochondrium and the distal one passing through the 12 mm suprapubic port. The 3 mm grasper in the right hypochondrium is also useful during hand suturing of the enterotomies.

Finally, attention must be paid when maneuvering 3 mm instruments, which must be done under direct vision throughout the operation.

Our experience suggests that in well-trained hands and for properly selected patients, ports can be reduced in size safely without a negative impact on the surgeon's ability to perform laparoscopic colorectal resections. These findings should promote a larger prospective randomized comparison with conventional laparoscopy to determine whether this refinement of laparoscopic colorectal surgery confers concrete and incontrovertible benefits to the patients.
